# A Comprehensive Review on the Efficacy of Antihypertensives in Patients With End‐Stage Renal Disease

**DOI:** 10.1155/ijhy/9930482

**Published:** 2026-07-21

**Authors:** Oluwakorede Akele, Nnedindu Asogwa, Gennifer Wahbah Makhoul, Khalil El-Gharib, Joanne Ling, Alaukika Agarwal, Vanessa Agberien, Ngowari Pokima, Thomas Gut, Suzanne El-Sayegh

**Affiliations:** ^1^ Department of Medicine, Northwell, New Hyde Park New York, 11042, USA, duhs.edu.pk; ^2^ Department of Medicine, Yale New Haven Health-Bridgeport Hospital, Bridgeport Connecticut, 06610, USA, bridgeporthospital.org; ^3^ Department of Nephrology, Northwell, New Hyde Park New York, 11042, USA

## Abstract

End‐stage renal disease (ESRD) patients are particularly susceptible to hypertension, as blood pressure (BP) dysregulation leads to increased mortality and morbidity, especially considering the heightened risk of cardiovascular complications, including myocardial infarction, stroke, and congestive heart failure. Given the lack of specific guidelines on therapies aimed at achieving adequate BP control in ESRD patients, we conducted a thorough literature review to identify relevant studies and articles exploring the associations of various therapies in ESRD patients and to provide a qualitative analysis based on these results. These include ACE inhibitors, ARBs, calcium channel blockers (CCBs), beta‐blockers, diuretics, alpha‐2 agonists, and direct vasodilators. ARBs are not affected by dialysis, while ACE inhibitors are dialyzable. Studies suggest that the incidence of cardiovascular events is significantly lower in ESRD patients receiving treatment compared to those without treatment. CCBs are nondialyzable and, thus, serve as a second‐line treatment for BP control in ESRD patients. Limited evidence suggests that diuretic‐based combination therapy may improve BP control in selected hemodialysis patients, but its effect on cardiovascular mortality remains uncertain. Beta‐blockers have demonstrated a favorable profile but differ in dialyzability and cardioselectivity, making it challenging to endorse an ideal choice of drug. Direct vasodilators have been shown to reduce left ventricular hypertrophy, but further studies are needed to determine optimal dosing strategies. Clonidine, an alpha‐2 agonist, lacks safety and efficacy evaluations but has been shown to reduce BP in ESRD patients.

## 1. Introduction

Hypertension, a prevalent condition characterized by persistently elevated blood pressure (BP), presents significant challenges in the management of patients undergoing dialysis. Individuals with end‐stage renal disease (ESRD) on hemodialysis (HD) are particularly vulnerable to the detrimental effects of hypertension, as impaired renal function further exacerbates BP dysregulation [1, 2]. The prevalence of hypertension in this population is concerning, with studies reporting rates as high as 86% [3].

The presence of hypertension in patients undergoing HD is associated with increased morbidity and mortality, primarily attributed to cardiovascular events [4, 5]. Cardiovascular disease is the leading cause of death in ESRD patients, responsible for 40% of cases, mainly due to myocardial infarction, vascular disease, and sudden cardiac death [6]. Multiple studies have shown that hypertension in HD patients is independently associated with increased risks of all‐cause mortality, cardiovascular mortality, and major adverse cardiovascular events [7, 8]. Furthermore, hypertension in HD patients has been linked to the development of left ventricular hypertrophy (LVH), a known marker of elevated cardiovascular risk. In a study by Levin et al., LVH was significantly more prevalent in HD patients with hypertension than those without hypertension [9]. Moreover, LVH has been associated with increased risk of cardiovascular events and mortality in ESRD patients [9]. Common cardiovascular risk factors such as high cholesterol, hypertension, and obesity do not fully account for the increased risk of cardiovascular death [10].

The pathogenesis of hypertension in ESRD involves volume expansion, activation of the renin–angiotensin–aldosterone system (RAAS), increased sympathetic nervous system activity, endothelial dysfunction, and arterial stiffness. In ESRD, the kidneys are unable to maintain sodium and water balance [11]. RAAS is activated in response to decreased renal perfusion, leading to sodium and water retention and resulting in volume expansion [12, 13]. Activation of RAAS also stimulates the release of angiotensin II, a potent vasoconstrictor. Angiotensin II promotes arterial stiffness by increasing collagen deposition and reducing elastin content in the arterial wall, thereby increasing peripheral resistance and contributing to hypertension [14].

In chronic kidney disease (CKD), signs and symptoms of volume overload develop early in the progression of the disease [15]. Volume overload, resulting from fluid retention and interdialytic weight gain, is directly associated with cardiovascular disease and serves as a prognostic indicator in HD patients [10, 16]. Fluid retention results from multiple factors, including decreased urine output, increased sodium intake, and reduced sodium excretion [17]. Increased sodium leads to increased extracellular volume, resulting in elevated BP. A lag phenomenon has been hypothesized in which BP normalization lags extracellular volume contraction following fluid management [18]. Volume overload can also cause endothelial dysfunction, impairing blood vessels′ ability to dilate and accommodate increased blood flow [15].

The intermittent, three‐times‐per‐week nature of HD predisposes patients to excessive weight gain between sessions and the excessive weight loss postdialysis, causing cardiovascular stress [19]. No objective methods are clearly defined to assess fluid overload, making optimal hydration in patients with ESRD difficult. Routine evaluation often relies on body weight and BP, but these do not accurately reflect actual volume status [20]. These limitations contribute to poor BP control and increase the risk of serious complications in this population.

The definition of hypertension in the HD population differs from that of the general population. According to the Kidney Disease Outcomes Quality Initiative (KDOQI) guidelines, hypertension in dialysis patients is defined as a predialysis systolic blood pressure (SBP) ≥ 140 mmHg or diastolic BP ≥ 90 mmHg, or a postdialysis BP > 130/80 mmHg [21]. However, the accuracy of peri‐dialytic BP readings is questionable, and staff recording practices may limit postdialytic measurements. These readings provide imprecise estimates of interdialytic BP compared to 44‐h ambulatory BP monitoring measurements [22].

The method of BP measurement is equally important, as this topic has also been debated. It is now widely recognized that managing hypertension solely based on predialysis BP targets is insufficient. Current recommendations favor using interdialytic home BP monitoring or calculating the mean or median of peri‐dialytic BP measurements [22]. However, median BP is easier to calculate than mean BP because of resource utilization [22], providing a more accurate assessment and better BP control [23].

Agarwal et al. demonstrated that incorporating intradialytic BP measurements alongside pre‐ and postdialysis readings improves diagnostic accuracy [22]. Ambulatory BP monitoring is considered the gold standard for diagnosing hypertension in ESRD patients and more accurately predicts all‐cause and cardiovascular outcomes compared to peri‐dialytic measurements [24, 25]. Nevertheless, it is crucial to recognize that the interpretation and management of hypertension in this population must account for comorbidities, individual patient characteristics, and dialysis‐specific factors.

Establishing appropriate BP goals is crucial for balancing the benefits of BP control with potential adverse effects, although recent guidelines have not stated a specific BP goal for HD patients [23]. Evidence suggests that the relationship between BP and cardiovascular mortality is “J”‐ or “U”‐shaped, indicating that excessively low predialysis or postdialysis diastolic or systolic pressures may increase cardiovascular and all‐cause mortality [4]. Interestingly, predialysis BP measurements were not associated with increased cardiovascular mortality, although postdialysis SBP > 180 mmHg and diastolic BP > 90 mmHg were associated with increased cardiovascular mortality [4].

A study showed that SBP < 110 mmHg was also associated with increased cardiovascular mortality, both pre‐ and postdialysis [4]. Pre‐ and postdialytic BP measurements limited this study. A study by Agarwal et al. demonstrated that better BP control, specifically lower SBP, was associated with a reduced risk of cardiovascular events and mortality in this population [26], stressing the importance of implementing aggressive BP management strategies in HD patients. Other studies have shown that the association between pre‐HD and peridialytic SBP with mortality is complex [27]. However, no large‐scale randomized controlled trial (RCT) has yet specifically addressed BP targets in this population.

The Japanese Society of Hypertension Guidelines for the Management of Hypertension 2019 recommend that patients with HD undergoing antithrombotic therapy have a BP target below 130/80 mmHg [28]. Supporting this, a study of patients undergoing HD found that favorable outcomes were associated with a predialysis SBP of 130–159 mmHg, a diastolic BP of 60–99 mmHg, and postdialysis systolic and diastolic BPs of 120–139 mmHg and 70–90 mmHg, respectively [29]. Although diastolic BP lacks substantial independent prognostic value, keeping it below 80 mmHg is generally advised [29]. In patients with a diastolic BP below 60 mmHg, systolic pressure should be kept above 140 mmHg [29]. The optimal therapeutic strategy in this population requires careful consideration of efficacy, safety, drug interactions, and potential adverse effects [30].

This comprehensive review article aims to provide a comprehensive overview of antihypertensive management in dialysis patients. By critically analyzing the existing literature, including clinical trials, meta‐analyses, and observational studies, we will examine the prevalence of hypertension in patients with HD, its associated morbidity and mortality, BP targets, and the specific challenges in defining and treating hypertension in this population. Additionally, we will assess the benefits and limitations of various antihypertensive drug classes in achieving BP control and reducing cardiovascular risk in dialysis patients.

## 2. Methods

A comprehensive search of PubMed, Web of Science, and Embase was conducted from database inception through January 2025 to identify relevant articles. The search strategy combined keywords and Medical Subject Headings (MeSH) terms, including “hypertension,” “end‐stage renal disease,” “hemodialysis,” “antihypertensive agents,” “high blood pressure,” “renal insufficiency,” “chronic kidney failure,” “cardiovascular disease,” “cardiovascular mortality,” and “renal replacement therapy.”

### 2.1. Inclusion and Exclusion Criteria

Studies were included in this review if they met the following criteria (1) conducted in patients with ESRD; (2) focused on antihypertensive treatment in ESRD patients; and (3) published in English. Studies involving nonhuman subjects, as well as opinion pieces, case reports, and editorials, were excluded.

### 2.2. Data Extraction and Analysis

Two reviewers independently screened the titles and abstracts of all identified studies to assess eligibility. They then obtained and reviewed full‐text articles of potentially eligible studies. Any discrepancies between reviewers were resolved through discussion and consensus. One reviewer extracted data and independently verified it by a second reviewer. Due to heterogeneity among the included studies, findings were summarized qualitatively.

## 3. Results and Discussion

### 3.1. Nonpharmacological Approaches for BP Control in HD Patients

Managing BP in ESRD patients undergoing HD presents unique challenges, prompting a thorough exploration of nonpharmacological strategies. A crucial initial step in hypertension management is achieving and maintaining an accurate dry weight, which depends on patient adherence to dialysis treatment and to volume and salt restriction guidelines. The impact of sodium intake on weight gain is emphasized, with a meta‐analysis by Cole et al. demonstrating a significant reduction of 8.4 mm Hg in SBP and a 4.4 mm Hg reduction in diastolic BP through dietary sodium restriction [31]. KDIGO guidelines recommend a sodium intake of less than 2 g per day, or less than 5 g of sodium chloride per day, for patients with CKD and high BP [32].

Monitoring dry weight involves various methods, including tracking weight trends, conducting physical examinations for signs of hypervolemia, and utilizing specific measurements such as cardiothoracic index (CTI), echocardiography, and bioimpedance in hypertensive patients [33]. A “strict volume control strategy” is proposed as the primary approach to hypertension management in dialysis patients, involving salt restriction, determination, and maintenance of dry weight, with CTI as a critical parameter.

While physical exercise is recognized as a potential strategy for managing hypertension, evidence regarding its effectiveness in patients undergoing maintenance HD remains limited and inconsistent, mainly due to small study populations and heterogeneous study designs [14]. Scapani et al. found that combined resistance and aerobic training reduced BP in patients with HD [34]. At the same time, other studies, such as those by Thompson et al., showed no significant impact [35]. Interdialytic exercise, which offers patient flexibility, is explored, but challenges such as postdialysis fatigue hinder consistent engagement. Future research is essential to assess the effects of exercise on BP and other outcomes in patients with HD.

Adjusting the sodium concentration in the dialyzate has been identified as a potential strategy to lower SBP. Sayarlioglu et al. proposed regulating dialyzate sodium concentration based on predialysis sodium levels, demonstrating a reduction in SBP and alleviating cardiac volume load, as assessed by echocardiography [36]. Inrig et al.′s study supports the notable impact of reducing dialyzate sodium concentrations, leading to a significant decrease in SBP and improvement in intradialytic hypertension [37]. Positive outcomes, including lowered pre‐ and postdialysis BPs, reduced ultrafiltration needs, and mitigated symptomatic intradialytic hypotension, were observed, accompanied by a slight reduction in dialyzate sodium, particularly in older patients and women, with limited effects in younger patients and lesser effects in men [38]. To advance our understanding, larger RCTs are warranted to evaluate strategies for optimizing dialyzate sodium prescriptions for patients undergoing maintenance HD.

### 3.2. Beta‐Blockers

Beta‐blockers are a well‐established class of medications used to manage various cardiovascular conditions, including heart failure, arrhythmias such as atrial fibrillation, and hypertension. They are considered essential in the treatment of heart‐related diseases. A notable meta‐analysis by Jin Guo and Yu demonstrated significant benefits of beta‐blocker therapy in patients with HD, including a 27% reduction in all‐cause mortality and a 56% reduction in cardiovascular mortality in RCTs [39]. While extensive research supports their effectiveness in the general cardiovascular population, evidence regarding their use in patients with ESRD is comparatively limited, highlighting an important area for further investigation.

A study demonstrating their underuse showed that only 8.5% of HD patients were treated with beta‐blockers [40]. This is concerning despite strong mechanistic rationale and observational evidence supporting their role in reducing cardiovascular morbidity and mortality [41]. Underutilization is primarily attributed to pharmacological heterogeneity within the drug class, adverse effect profiles such as hypotension and hyperkalemia, and the lack of large‐scale randomized trials in the ESRD population [41]. Evidence‐based recommendations favor carvedilol for patients with heart failure, hypertension, ischemic heart disease, or those at risk for sudden cardiac death, with atenolol and bisoprolol as alternative agents based on dialytic clearance and dosing feasibility [41].

The challenge with this drug class lies in determining which agent is most appropriate for this unique patient population. It is crucial to categorize beta‐blockers by dialyzability and cardioselectivity to address this. Haddiya and Valoti introduced the concept of considering both properties when choosing beta‐blockers for ESRD patients [42]. Dialyzability refers to a drug’s ability to be effectively removed from the body during dialysis [43], which plays a pivotal role in determining the suitability of a beta‐blocker for patients undergoing HD [43]. For example, drugs like atenolol and metoprolol are highly dialyzable, meaning they can be efficiently eliminated during dialysis sessions [43]. This can, in turn, lead to rebound hypertension in the postdialysis period [44]. In contrast, drugs like carvedilol and propranolol are less readily removed, making them less suitable choices for ESRD patients [43]. Notably, the highly dialyzable drugs used in most studies are also recognized for their cardioselective characteristics [42].

A notable study by Tella et al. conducted a meta‐analysis comparing the efficacy of highly dialyzable versus poorly dialyzable beta‐blockers with respect to cardiovascular events and mortality among HD patients [45]. The study found that highly dialyzable beta‐blockers had a similar mortality rate compared to poorly dialyzable agents (HR: 0.94, 95% CI: 0.81–1.08) [45]. However, highly dialyzable drugs were associated with a significant reduction in cardiovascular events (HR 0.88, 95% CI: 0.83–0.93) [45]. Notably, the results did not support the use of carvedilol, which has a lower clearance rate during dialysis [45].

In contrast to these findings, a retrospective cohort study of 6588 elderly U.S. dialysis patients reported that those receiving highly dialyzable beta‐blockers had a 40% higher risk of mortality compared to those on poorly dialyzable agents (RR 1.40, 95% CI: 1.1–1.8) [46]. Over 6 months, mortality was 5.5% in the dialyzable group versus 4.1% in the nondialyzable group, and a modest increase was also seen in a composite outcome of death, MI, or heart failure [46]. These findings raise concerns that beta‐blockers removed during dialysis may provide less consistent cardioprotection, particularly as clinicians did not adjust doses to compensate and medication levels remained stable in both groups.

An extensive Taiwanese cohort study of approximately 36,600 new HD patients found that those started on dialyzable beta‐blockers had slightly lower 2‐year mortality compared to those on nondialyzable agents (adjusted HR: 0.82, 95% CI: 0.75–0.88) [43]. They also had a lower risk of major cardiovascular events, including myocardial infarction, stroke, and heart failure (HR: 0.89, 95% confidence interval 0.84–0.93) [43]. These findings remained consistent after propensity score matching and sensitivity analyses. The authors suggested that concerns about dialyzability may be overstated and emphasized the importance of considering patient context when assessing the effectiveness of beta‐blockers in dialysis patients.

In a pivotal study, Bakris, Hart, and Ritz compared carvedilol to placebo in HD patients, finding a decrease in all‐cause mortality among those with cardiomyopathy who received carvedilol [47]. However, the study’s limitations become evident when carvedilol is compared to more dialyzable beta‐blockers [47]. Studies indicate that HD patients with heart failure can benefit from beta‐blocker therapy. For example, in a 10‐year cohort of dialysis patients with systolic heart failure, use of carvedilol, bisoprolol, or extended‐release metoprolol was associated with improved survival compared with patients without antihypertensive therapy [48].

In a 2‐year randomized trial of HD patients with heart failure, carvedilol significantly reduced all‐cause mortality to 51.7% compared to 73.2% with placebo (*p* < 0.01) [49]. Cardiovascular deaths (29.3% vs. 67.9%) and heart failure hospitalizations (34.5% vs. 58.9%) were also markedly lower in the carvedilol group compared to placebo (*p* < 0.00001), supporting its role in improving outcomes in this high‐risk population [49]. A multicenter pilot trial demonstrated limited tolerability of beta‐blockers such as carvedilol: only 68% of patients completed the run‐in phase without bradycardia or hypotension, and more withdrawals occurred in the carvedilol group (4 of 26) than in the placebo group (0 of 23) [50]. Although intradialytic hypotension was more frequent with carvedilol (7 vs. 2 episodes per 100 sessions, *p* = 0.10), the study was underpowered to assess clinical outcomes [50].

The HDAPL trial, which involved 200 patients on maintenance HD with echocardiographic evidence of LVH and hypertension, reduced left ventricular mass index (LVMI) [51]. Initially, the lisinopril and atenolol groups exhibited similar 44‐h ambulatory BP measurements, but after 12 months, atenolol showed a numerically greater reduction in BP based on 44‐h interdialytic ambulatory BP monitoring (mean reduction of 21/13 mmHg vs. 18/10 mmHg, respectively) and self‐measured home BP readings (mean reduction of 25/12 mmHg vs. 19/10 mmHg, respectively) compared to lisinopril [51]. The trial was terminated prematurely because of the significant superiority of atenolol over lisinopril in preventing adverse cardiovascular outcomes [51]. Lisinopril was associated with an elevated risk of hospitalizations for congestive heart failure, all‐cause hospitalizations, cardiovascular morbidity, and the combined risk of hospitalizations due to heart failure, myocardial infarction, stroke, and cardiovascular death [51]. Additionally, lisinopril‐based therapy was linked to an increased risk of hyperkalemia and emergent treatment for hypertensive crises [51].

The use of beta‐blockers is not without risk. Evidence has shown that beta‐blocker use in HD patients is associated with bradycardia and hypotension, particularly with nonselective agents. The BLOCADE trial demonstrated that the risk of intradialytic hypotension may be elevated with vasodilating beta‐blockers such as carvedilol [50]. Beta‐blockers can also blunt the tachycardia response to hypovolemia, potentially worsening intradialytic hypotension.

While these studies provide valuable insights, it is essential to recognize that the specific beta‐blocker used often limits the findings. Each agent in this class has distinct pharmacokinetic properties, making it challenging to determine the optimal drug for cardiovascular benefit in patients with HD. Therefore, further research is needed to identify the most effective beta‐blockers for this patient population.

### 3.3. ACE Inhibitors and Angiotensin Receptor Blockers (ARBs)

One proposed mechanism for the development of systemic hypertension in ESRD is increased activity of the RAAS [52]. Current guidelines recommend angiotensin converting enzyme inhibitors (ACEIs) or ARBs as first‐line agents for patients with ESRD, particularly those with diabetes mellitus [53]. A large‐scale, population‐based cohort study from Taiwan demonstrated that ESRD patients using ACEIs or ARBs had significantly lower all‐cause mortality than non‐users, with an adjusted hazard ratio of 0.47 (95% CI: 0.46–0.49) [54]. This is further supported by a retrospective analysis from 2002 examining ACEI use in HD patients, which reported a significant 52% reduction in mortality risk (RR 0.482, 95% CI 0.25 to 0.91, *p* < 0.0019) in the treated group compared to untreated patients [55]. The reduction was even more pronounced in patients 65 years or younger, with a 79% absolute risk reduction (RR 0.211, 95% CI: 0.08–0.58, *p* < 0.0006) [55]. These findings suggest that age may be an essential factor in determining the benefits of these medications. Other studies also indicate that RAAS inhibition therapy may reduce cardiovascular events, although current guidelines do not clearly delineate whether to continue or discontinue therapy upon the initiation of HD [56].

In an observational analysis of HD patients, a comparison of the relative efficacy of ACEIs and ARBs found no significant difference in cardiovascular mortality between patients initiated on either medication class (HR 0.96) [57]. The same study also compared combination therapy with ACEIs and ARBs to therapy combining an ACEI or ARB with a non‐ACEI/ARB antihypertensive agent and found that the combination of an ACEI and ARB was associated with a significantly increased risk of cardiovascular death [57]. The study suggested that ARB‐based therapy might offer a safer cardiovascular profile in this patient population, as the hazard ratio for ARB plus another antihypertensive agent was 0.69 (95% CI: 0.57–0.84), compared to 0.87 (95% CI: 0.72–1.06) for the ACEI plus other antihypertensive groups [57].

In contrast, Cice et al. studied 332 Italian HD patients with congestive heart failure and left ventricular ejection fraction (LVEF) < 40%, showing that adding telmisartan (80 mg/day) to ACE inhibitor therapy over 35.5 months reduced all‐cause mortality by 49%, cardiovascular mortality by 58%, and CHF hospitalization by 62% [58]. Although a multimodal strategy using dual therapy, BP control, statins, and lifestyle changes safely induced remission or regression of proteinuria and stabilized kidney function in CKD patients with heavy proteinuria, nearly preventing ESRD progression [59], no studies have specifically explored dual therapy in HD patients, leaving the available data inconclusive.

Evidence shows that BP reduction is not significantly different between ACEI‐treated and untreated dialysis patients, despite a significant mortality benefit in the treated group [55]. Earlier trials suggested that discontinuing ACEIs or ARBs in this population might improve kidney function, with reported increases in estimated glomerular filtration rate (eGFR) following withdrawal [60]. However, more recent studies have demonstrated that discontinuing RAS inhibitors did not lead to meaningful improvements in eGFR, either overall or within key subgroups, and rates of kidney failure, cardiovascular events, and death were similar between groups over 3 years [56]. Although the discontinuation group initially showed higher BP and proteinuria during the first year, these differences diminished with the use of other antihypertensive therapies [56]. These findings suggest that the benefits of ACEIs in this population may extend beyond their antihypertensive effects. The benefits of ACEI in dialysis patients may not be solely due to their impact on BP [55]. While ACEI and ARB treatment produced BP changes similar to those in controls, it was still associated with improved outcomes, including reduced mortality and fewer heart failure events [61].

A retrospective cohort study using the medication possession ratio method to assess adherence found that patients with poor adherence to ACEIs or ARBs had a lower adjusted mortality risk (HR: 0.81, 95% CI: 0.76–0.86) compared to non‐users, while those with good adherence showed no significant mortality benefit (HR: 0.98, 95% CI: 0.86–1.13) [62]. This counterintuitive finding raises questions about potential confounding factors in observational studies. Further support comes from a meta‐analysis that found that ACEIs and ARBs did not significantly reduce cardiovascular events, cardiovascular mortality, or all‐cause mortality compared with placebo or no additional treatment [63]. These findings were consistent across subgroups defined by hypertension status, ethnicity, study size, follow‐up duration, or trial quality.

In the FOSIDIAL trial, fosinopril did not significantly reduce the risk of cardiovascular events compared to placebo in the intention‐to‐treat analysis [64]. However, a nonsignificant trend toward benefit was observed in the per‐protocol analysis [64]. Among patients who were hypertensive at baseline, fosinopril led to significantly greater reductions in both systolic and diastolic BP compared to placebo, while no significant differences were observed in normotensive patients [64]. In the same year, Takahashi and colleagues conducted a study of 80 patients undergoing HD over 19 months, finding that candesartan significantly reduced cardiovascular events (16% vs. 46% in controls) and improved survival (0% vs. 19% mortality) [65]. No deaths occurred in the ABR group, whereas mortality was 18.9% in controls. It was associated with better cardiovascular outcomes, with no reports of severe hyperkalemia or hypotension [65].

In a secondary analysis of the HEMO study examining ACEI use in HD patients, ACEI use was associated with a higher risk of heart failure hospitalization in multivariable‐adjusted analyses (HR: 1.41, 95% CI: 1.11–1.80) [66]. However, this association was no longer observed after adjusting for baseline differences, suggesting that the original result may have been affected by unaccounted‐for factors. In contrast, a meta‐analysis specifically focused on ARB therapy found that it reduced the risk of heart failure events by 33% (RR: 0.67, 95% CI: 0.47–0.93) in dialysis patients, with BP reductions comparable to those observed with placebo or another active agent [61]. This finding suggests that ARBs may offer specific benefits for heart failure outcomes in this population. A multicenter open‐label trial (366 patients screened; 180 randomized to ARB therapy and 180 to control after a run‐in period) comparing ARB therapy (valsartan, candesartan, or losartan) to no ARB use in HD patients over approximately three years of follow‐up found that ARB therapy reduced the risk of cardiovascular events by half. In this trial, 19% of ARB‐treated patients experienced a fatal or nonfatal cardiovascular event compared to 33% of control patients (adjusted HR: 0.51, 95% CI: 0.33 to 0.79, *p* = 0.002) [67].

ACEIs and ARBs can increase the risk of hyperkalemia, which is particularly concerning in ESRD patients who already have impaired potassium excretion. One study noted that the adverse effects of ACEIs and ARBs, including hyperkalemia, are more common in patients with CKD and ESRD [62]. Interestingly, the FOSIDIAL trial did not find a high incidence of hyperkalemia attributed to ACEIs or ABRs [64]. The HELD trial, a single‐blind study involving 88 HD patients, found that losartan modestly but significantly lowered postdialysis SBP over eight weeks without causing an increased incidence of intradialytic hypotension or hyperkalemia [68]. Patients in the losartan group achieved better BP control than those receiving standard therapy, with no significant side effects reported [68]. Intradialytic hypotension affects up to 30% of dialysis sessions and is associated with complications such as poor dialysis tolerance and vascular access problems [69]. Because HD itself can cause significant BP fluctuations leading to symptoms, reduced dialysis efficiency, and increased cardiovascular risk, clinicians often adjust or withhold antihypertensive medications and may administer vasoconstrictors during treatment. Regarding dialyzability, ARBs are unaffected by dialysis, whereas ACEIs are dialyzable [44]. This pharmacokinetic difference provides a theoretical advantage for ARBs in maintaining consistent drug levels in this population.

The evidence regarding the use of ACEIs and ARBs in HD patients remains mixed. These discrepancies highlight significant gaps in the current literature and emphasize the need for large, well‐designed RCTs specifically targeting the HD population. Until more definitive evidence becomes available, treatment decisions regarding ACEIs and ARBs in HD patients should be individualized, taking into account each patient’s cardiovascular risk profile, BP control requirements, tolerance to these medications, and potential for adverse effects.

### 3.4. Calcium Channel Blocker (CCB)

CCBs are the most commonly prescribed drug class for HD patients [40] and are used to manage hypertension, angina, and supraventricular tachycardia. Youssef et al. showed that amlodipine reduces LVMI more than bisoprolol (−35 g/m^2^ vs. −9.8 g/m^2^, *p* = 0.017) [70]. These findings, along with improvements in endothelial function markers, suggest that CCBs may offer superior cardiovascular protection in hypertensive HD patients and warrant consideration as a preferred first‐line option.

There are two subclasses of CCBs: dihydropyridines (DHPs) and non‐dihydropyridines (non‐DHPs), which primarily target vascular smooth muscle and the myocardium, respectively [71]. Both subclasses are effective in lowering BP in patients with ESRD. Due to their favorable pharmacokinetics and nondialyzable properties, DHPs are preferred as second‐line agents for BP control in dialysis patients [72].

A small RCT involving 40 HD patients, divided into verapamil and amlodipine groups, showed that BP fell substantially in both groups (approximately 21/23 mmHg with amlodipine and 17/21 mmHg with verapamil) [73]. Amlodipine normalized diastolic BP (≤ 90 mmHg) in 81% of patients compared to 74% with verapamil, although the difference was not statistically significant [73]. A more recent cohort study comparing DHP and non‐DHP CCBs found that DHP CCB use was associated with better cardiovascular outcomes, with a lower hazard for the combined endpoint of cardiovascular morbidity or mortality (adjusted HR approximately 0.77 compared to non‐DHP, *p* < 0.001) [74].

Studies have shown that DHPs, such as amlodipine, can significantly reduce all‐cause mortality and cardiovascular events in HD patients. A double‐blind trial conducted by Tepel et al. randomized 251 hypertensive HD patients into two groups: one receiving oral amlodipine 10 mg daily and the other receiving a placebo, with a median follow‐up of 19 months [75]. Although amlodipine was associated with a nonsignificant improvement in overall survival, it reduced all‐cause mortality and cardiovascular events by 47% in hypertensive patients [75]. Additionally, amlodipine significantly reduced the composite outcome of mortality and nonfatal cardiovascular events, with 15% of patients in the amlodipine group reaching the endpoint compared to 25% in the placebo group (HR: 0.53, 95% CI: 0.31 to 0.93, *p* = 0.03) [76]. In a national U.S. cohort of 3716 incident HD patients, those prescribed a CCB had a significantly lower risk of cardiovascular death over 2 years. CCB users had a 26% lower CARDIOVASCULAR mortality rate than non‐users (RR: 0.74, 95% CI: 0.60–0.91) [74]. The same study also showed that CCB users had a 21% lower risk of all‐cause mortality after adjustment for confounders (RR 0.79, 95% CI 0.69–0.90) [74].

When compared with ACEIs or ARBs, CCBs achieve comparable BP control in patients with ESRD. A meta‐analysis of 21 RCTs in CKD stage 3 to 5D (including dialysis patients) reported no significant difference in BP reduction between CCB therapy and RAS blockade [77]. This is further supported by Yilmaz et al., who found that amlodipine and ramipril achieved equivalent BP control in nondiabetic HD patients [78], further supporting this.

Intradialytic hypotension is a potential concern in patients taking CCBs; however, evidence suggests this risk may be limited. The incidence of symptomatic hypotension during dialysis was not increased with amlodipine compared to placebo (RR 0.54, 95% CI 0.25–1.15) [76]. Overall, CCBs appear safe with respect to intradialytic hypotension, particularly when dosed appropriately. Some CCBs may also offer benefits for specific BP management challenges. An open‐label multicenter trial of cilnidipine in patients with intradialytic hypertension demonstrated improved ambulatory BP control and reduced BP variability [76]. Although data are limited, these findings suggest that newer CCBs targeting multiple calcium channel subtypes can aid in stabilizing BP, even in challenging scenarios.

DHPs are known to cause peripheral edema due to precapillary vasodilation. Dialysis patients are already prone to fluid retention, so edema is a significant side effect. The incidence of this is low and manageable in the HD population [73]. Non‐DHP CCBs were well tolerated in ESRD, with no serious bradyarrhythmias reported, though higher doses of verapamil occasionally caused reflex tachycardia [73]. Caution is advised in patients with bradycardia or on beta‐blockers. DHPs, such as amlodipine, did not significantly affect heart rate in dialysis patients; overall, both CCB types showed a favorable safety profile, with no excess arrhythmias or ischemic events [73].

Small study sizes, short follow‐up periods, and inconsistent reporting of outcomes limit the current evidence on hypertension management in HD. Larger, well‐powered trials with standardized methods and longer follow‐up are needed, especially in high‐risk subgroups like those with heart failure or coronary disease.

### 3.5. Diuretics

Several studies have investigated the effect of diuretics on BP control in patients with HD. Diuretic therapy has been associated with improved BP control, with a significant reduction in SBP observed in patients receiving diuretics compared to those who did not [79]. Combining diuretics with other antihypertensive agents has resulted in greater BP reduction than monotherapy in patients with HD, suggesting additive or synergistic effects [80]. Despite their potential benefits, there are inconsistencies regarding the continued use of diuretics in HD patients. No dedicated trials have assessed adverse cardiovascular events or mortality in patients with HD. Prior studies tend to lump all diuretic users together, which has been shown to lower cardiac mortality [81]. Although diuretic use has declined in the United States, it remains more common in Europe and Asia [82].

Due to their pharmacokinetic properties, loop diuretics exhibit limited dialyzability because they are highly protein‐bound (approximately 90%), which restricts their removal during HD. After all, protein‐bound molecules are not efficiently filtered through dialysis membranes [83]. Loop diuretics are often discontinued at dialysis initiation under the assumption that they are ineffective in anuric patients [84]. However, loop diuretic use in patients with residual renal function has been associated with improved survival rates. For example, in the DOPPS study, patients receiving diuretics had a 14% lower risk of cardiac death (*p* = 0.03) and a nonsignificant 7% lower risk of all‐cause mortality compared to non‐users [81]. Similarly, a 2019 cohort study found no significant difference in 1‐year mortality between those who continued versus discontinued loop diuretics at dialysis initiation (adjusted HR: ∼ 0.92, 95% CI 0.84 to 1.01 [85]), suggesting no harm and possibly a modest benefit.

These findings should be interpreted cautiously. Diuretics are more commonly continued in patients with preserved residual urine output, and residual kidney function is independently associated with lower mortality and better cardiovascular outcomes in patients with HD. Therefore, observed benefits in observational studies may reflect, at least in part, confounding by baseline residual kidney function rather than a direct causal effect of diuretic therapy. Randomized studies are needed to better define the treatment effect.

One study showed that higher doses of furosemide could increase urine volume by more than 60% over a year, although the response diminished over time [86]. Improved volume status may also reduce cardiac strain, as the same study reported significantly lower average interdialytic weight gain among loop diuretic users [85]. Even in ESRD, loop diuretics can lead to volume depletion and electrolyte disturbances if urine output remains significant. Care must be taken to avoid dehydration and hypotension, especially when combined with ultrafiltration. However, patients who continued diuretics experienced fewer intradialytic hypotension episodes [85], likely because they came into sessions less volume overloaded.

Thiazide diuretics are ineffective once the GFR is below 45 mL/min [87]. A 2023 meta‐analysis of trials in patients with CKD stages 3b, 4, or 5 found that thiazides significantly reduce BP even at very low GFR [88]. Thiazide diuretics are not easily removed during HD due to their high protein binding and large volume of distribution, which limit their dialyzability. This allows for standard dosing in HD patients [89]. It is important to note that thiazide diuretics differ in metabolism and excretion. For example, hydrochlorothiazide has a shorter half‐life (6–15 h) and dose proportionality, while chlorthalidone has a much longer half‐life (40–60 h), extensive red blood cell partitioning, and gradual plasma elimination [90].

Thiazides can cause hyponatremia and hypokalemia, although dialysis patients are somewhat protected from severe hypokalemia because ESRD often entails reduced potassium excretion [91]. Still, cases of thiazide‐induced hypokalemia have been noted in those with residual function. Volume depletion and hypotension can occur if diuresis is significant; careful titration is required to avoid exceeding the dry weight.

Diuretics enhance urine output, potentially reducing interdialytic weight gain. However, the optimal dosing strategy for diuretics in patients with HD remains unclear, and evidence on the most effective regimen is limited [82]. Diuretics promote urine output, improve volume control, and enhance electrolyte excretion in patients with HD [85]. This effect is significant for patients with residual urine output, as diuretics can help maintain consistent volume management and assist in electrolyte removal.

While diuretics have shown promise in BP control and volume management in HD patients, further research is necessary to establish optimal dosing strategies, evaluate long‐term outcomes, and assess their impact on cardiovascular morbidity and mortality in this specific patient population.

### 3.6. Direct Vasodilators

Many studies have shown that hydralazine and isosorbide nitrates are excellent medications, particularly in patients with resistant or refractory hypertension [92]. Hydralazine is a direct‐acting arterial vasodilator that reduces peripheral resistance and BP. Although limited data specifically on hydralazine use in HD patients exist, some studies have investigated its antihypertensive effects in patients with renal disease. HD does not quickly clear hydralazine due to its extensive first‐pass metabolism and strong protein‐binding capacity [93]. Although isosorbide dinitrate undergoes hepatic metabolism, its poor protein‐binding capacity makes it easily dialyzable [93].

Charytan et al. compared combination therapy with hydralazine and isosorbide dinitrate in dialysis‐dependent ESRD patients to a placebo [94]. The trial’s primary outcome was the change in LVMI from baseline to 12 months [94]. Secondary outcomes included changes in other cardiac measures, BP control, and adverse events [94]. The study’s results indicated that the combination therapy of hydralazine and isosorbide dinitrate did not significantly reduce LVMI compared to placebo after 12 months [94]. Additionally, there were no significant differences in secondary outcomes, including BP control and adverse events, between the treatment and placebo groups [94]. Another study reported favorable outcomes regarding BP reduction and a significant decrease in LVH but used only isosorbide [95]. In this prospective trial conducted in China involving 144 patients with HD, treatment with isosorbide mononitrate for 24 weeks reduced LVH and decreased the incidence of acute heart failure compared with placebo [95].

Mavrakanas et al. conducted a retrospective analysis of patients with ESRD on dialysis who were prescribed hydralazine and isosorbide dinitrate [93]. The study’s primary outcome was the change in BP after medication initiation [93]. Secondary outcomes included adverse events, hospitalizations, and mortality rates [93]. The results indicated that the use of hydralazine and isosorbide dinitrate in patients with ESRD on dialysis significantly reduced BP [93]. They also found that H‐ISDN therapy may improve cardiovascular outcomes in maintenance dialysis patients with heart failure [93]. However, the study also revealed an increased risk of adverse events, including symptomatic hypotension and worsening renal function, in some patients [93].

While hydralazine has demonstrated efficacy in reducing BP, potential adverse effects and individual patient characteristics must be considered. Hydralazine can cause reflex tachycardia, fluid retention, and lupus‐like syndrome in some individuals. Careful monitoring and appropriate dose adjustments are necessary to minimize these risks.

These studies provide important insights into the use of hydralazine and isosorbide dinitrate combination therapy in dialysis‐dependent ESRD patients. However, further large‐scale studies are needed to determine the efficacy, optimal dosing strategies, long‐term benefits, and safety of this combination therapy in this specific patient population. Additionally, comparative studies assessing the efficacy and safety of hydralazine versus other commonly used antihypertensive agents in this population would provide valuable insights.

### 3.7. Alpha‐2 Agonist (Clonidine)

Clonidine, an imidazoline derivative, has been frequently used to treat hypertension across diverse patient groups. It acts as an alpha‐2 adrenergic agonist in the nucleus tractus solitarius and as an antagonist in the medulla, thereby decreasing sympathetic signals and reducing arterial BP [96]. Despite its frequent use, clonidine’s efficacy is limited in ESRD patients. Approximately 40%–50% of the drug is excreted by the kidneys, necessitating lower dosing in ESRD patients [97, 98]. Additionally, Clonidine is dialyzable, which can cause fluctuations in BP. It often causes central nervous system side effects, such as drowsiness, dry mouth, and orthostatic hypotension, making it a last‐resort option [96].

Derk et al. reviewed eight studies on clonidine use in adult patients with HD; however, the total sample size was only 24, limiting the strength of the evidence [99]. Short‐term clonidine therapy (2–12 weeks) significantly reduced SBP by approximately 13 mmHg, while changes in diastolic BP were not statistically significant [99]. Side effects were common, including hypotension, drowsiness, and rebound hypertension. No long‐term cardiovascular or mortality data were available, and the authors cautioned against prolonged use due to potential risks, including fluid overload. Clonidine may lower BP acutely, but its long‐term safety in ESRD remains unclear.

Transdermal clonidine has shown benefit in cases of nonadherence among dialysis patients, as it offers a simpler mode of administration and requires less frequent dosing. A small crossover trial compared weekly transdermal clonidine patches (0.1–0.3 mg) with conventional oral therapy across two 6‐week periods [100]. However, no formal recommendations are currently available for the use of transdermal clonidine as first‐line therapy in ESRD patients with hypertension.

Other centrally acting alpha‐2 adrenergic agonists, including methyldopa and guanfacine, are now infrequently used for chronic hypertension management due to poor tolerability and a high burden of adverse effects [92]. Manufacturer labeling for methyldopa indicates that the drug is substantially cleared during HD, with reports of postdialysis recurrence of hypertension. This pharmacokinetic characteristic limits its clinical utility in dialysis‐dependent patients, as the dialysis procedure itself may undermine sustained BP control [101].

Guanfacine, a selective alpha‐2A adrenergic agonist, has been studied as an antihypertensive agent, with evidence of reductions in systolic and diastolic BP in trials of essential hypertension. Pharmacokinetic studies in patients with terminal renal failure indicate low dialysis clearance, suggesting that guanfacine exposure is relatively unaffected by HD and dose adjustments around dialysis sessions may not be necessary [102]. Additionally, renal impairment appears to alter guanfacine elimination without significant accumulation, as reported modestly. While direct evidence in HD cohorts remains limited, narrative reviews affirm its role as a centrally acting adjunctive agent in resistant hypertension in CKD and dialysis patients [103].

### 3.8. Mineralocorticoid Receptor Antagonist (MRAs)

MRAs, such as spironolactone, are commonly prescribed for nondialysis patients with resistant hypertension. However, their use is generally avoided in HD patients due to the potential risk of hyperkalemia. MRAs are primarily metabolized by the liver and exhibit high protein binding, making them poorly dialyzable.

Studies such as the Dialysis Outcomes Heart Failure Aldactone Study by Matsumoto et al. demonstrated that spironolactone reduces the risk of cardiovascular and cerebrovascular morbidity and mortality in HD patients, with a low incidence of hyperkalemia (1.9%) [104]. A RCT by Lin et al. further supported these findings, showing a significantly lower occurrence of deaths from cardiovascular events or any cause in the spironolactone group [105]. Parameters such as LVMI, LVEF, ventricular ejection fraction (VEF), and the number of antihypertensive drugs showed greater improvement in the spironolactone group [105]. Notably, no participants discontinued the study due to hyperkalemia [105].

In a 36‐week, placebo‐controlled trial of 129 patients with HD, spironolactone at 12.5–50 mg daily was generally safe and well tolerated [106]. However, spironolactone did not improve diastolic function or other cardiac parameters, suggesting no short‐term cardiovascular benefit in this modestly sized trial [106]. Nearly all participants completed dose escalation, and serious adverse events, including hyperkalemia and hypotension, were rare and comparable to placebo. Although the highest dose showed a slight trend toward increased hyperkalemia, overall event rates were similar between groups [106]. This is further supported by another RCT, which showed that 25 mg of spironolactone significantly reduced BP without causing severe hyperkalemia [107]. These findings suggest that spironolactone is a safe and effective short‐term option, although long‐term cardiovascular outcomes were not assessed. Although hyperkalemia is a significant concern with MRAs, especially in HD patients, multiple studies have shown that hyperkalemia events are minimal and comparable to placebo [108, 109].

Regarding cardiovascular outcomes, a daily dose of 50 mg of spironolactone did not improve left ventricular mass or other measures of cardiac function. While moderate hyperkalemia was more frequent, there was no increase in severe events, and the drug was generally well tolerated. No deaths occurred in the spironolactone group, although the study was not powered to assess mortality [110].

Finerenone is a new, selective, nonsteroidal MRA that has been shown to improve cardiac and renal outcomes in patients with CKD; however, patients with advanced disease are often excluded from RCTs due to the risk of hyperkalemia [111]. In a retrospective study of patients with Type 2 diabetes and advanced CKD (eGFR < 25 mL/min/1.73 m^2^), finerenone 10 mg daily significantly slowed the rate of eGFR decline, improving from −7.63 ± 9.84 to −1.44 ± 3.17 mL/min/1.73 m^2^ over six months (*p* = 0.038) [112]. Finerenone did not significantly increase serum potassium (*p* = 0.097) or alter SBP (*p* = 0.255). These findings suggest that finerenone may offer renoprotective benefits in advanced diabetic kidney disease without increasing the risk of hyperkalemia or affecting BP, supporting its potential use in more severe stages of CKD beyond those included in prior trials [112].

In a 13‐week multicenter trial of 154 HD patients, eplerenone (up to 50 mg daily) was well tolerated, with a lower rate of treatment discontinuation due to hyperkalemia or hypotension (4.0% vs. 2.8% with placebo) [113]. Although eplerenone was associated with more episodes of potassium levels greater than 6.5 mEq/L, these events were managed without increased dropout. BP and short‐term cardiovascular outcomes remained unchanged, supporting the short‐term safety of eplerenone in closely monitored ESRD patients [113].

### 3.9. Minoxidil

Initially developed for the treatment of alopecia, minoxidil is now being explored as an adjunct therapy for hypertension management in HD patients, particularly in cases of resistant hypertension where standard therapies are insufficient. Minoxidil has a large volume of distribution but lacks protein binding capacity, making it readily dialyzable [114]. Mundt et al. observed a significant reduction in BP with minoxidil administration, from 162.4 ± 15.1/83.2 ± 12.7 mm Hg to 135.8 ± 12.2/72.8 ± 6.9 mm Hg (*p* < 0.0001) [115]. This reduction remained consistent across subgroups, including ESRD patients [115]. Similarly, Schacht et al.′s study, which included patients with ESRD, reported a substantial reduction in BP [116].

In an observational study of 58 patients with advanced renal failure, long‐term minoxidil therapy significantly reduced BP from an average of 205/120 mmHg to 150/80 mmHg (*p* < 0.001) and allowed for simplified antihypertensive regimens [117]. Common side effects included hypertrichosis and fluid retention, which were managed with beta‐blockers and diuretics [117]. Notably, five of 11 patients with malignant hypertension recovered sufficient renal function to discontinue dialysis. These findings support minoxidil as a potent option for refractory hypertension in ESRD, with careful monitoring [117].

Despite its efficacy, minoxidil is associated with potential adverse effects, including fluid retention, tachycardia, hypertrichosis, and dermatological reactions [116]. Caution is advised when prescribing minoxidil to patients at risk of volume overload, who require close monitoring of fluid status, electrolytes, and heart rate during therapy [116]. While minoxidil holds promise as an adjunctive antihypertensive agent for patients monitored with HD due to its vasodilatory properties, careful patient selection and vigilant management of potential side effects are essential to optimize its use.

## 4. Conclusion

Hypertension poses significant challenges in patients with ESRD undergoing HD. The high rates of cardiovascular morbidity and mortality in this population underscore the need for targeted BP management. However, optimal antihypertensive strategies remain undefined due to insufficient evidence.

ARBs and ACE inhibitors have demonstrated cardiovascular benefits but are limited by dialyzability and potential adverse events. CCBs, such as amlodipine, are emerging as safer second‐line alternatives. Combination therapy with diuretics shows promise for additive BP reduction. The selection of beta‐blockers remains complicated by variable dialyzability and cardioselectivity. Direct vasodilators, including hydralazine and isosorbide, may improve LVH but require cautious dosing due to associated risks. Although widely used for BP control, alpha‐agonists such as clonidine lack robust efficacy and safety data in patients with ESRD (Table 1).

**TABLE 1:** Summary of antihypertensive therapies.

Drug class	Medication	Dialyzability	Efficacy	Mortality impact	References
Beta‐blockers	Carvedilol Atenolol Metoprolol Bisoprolol Propranolol	Selective beta‐blockers (metoprolol, atenolol, bisoprolol): highly dialyzable Nonselective beta‐blockers (carvedilol, propranolol): poorly dialyzable	Effective in lowering BP Effective in patients with systolic heart failure, ischemic heart disease, and arrhythmias Effective, but selection depends on dialyzability and cardioselectivity Variability in beta‐blocker pharmacokinetics makes it challenging to determine the single most effective option for cardiovascular protection Increased risk of intradialytic hypotension and blunt compensatory tachycardia during hypovolemia.	Highly dialyzable beta‐blockers significantly reduced cardiovascular events but showed no mortality difference compared to poorly dialyzable agents. Reduces all‐cause mortality, but mortality impact varies by agent and dialysis clearance rate, with inconsistent results across observational studies. Careful timing and dosing to prevent rebound hypertension in highly dialyzable drugs	[39, 41–51, 70]

ACEIs	Lisinopril, ramipril, fosinopril	Dialyzable	Effectively lowers BP in ESRD patients, particularly those with diabetes, but they have limitations May offer cardiovascular protection beyond their BP‐lowering effects Benefits of ACEIs in HD patients may be influenced by patient age, comorbidities, and the degree of volume control ACEIs are associated with the risk of hyperkalemia and increased hospitalizations compared to beta blockers.	The combination of ACEIs and ARBs increases cardiovascular risks compared to monotherapy, suggesting monotherapy is safer The FOSIDIAL trial showed that fosinopril significantly reduced systolic and diastolic BP in hypertensive dialysis patients compared to placebo, although it did not significantly reduce cardiovascular events overall The effectiveness of ACEI in reducing cardiovascular events shows mixed results. Some studies suggest they are not superior to placebo in reducing CARDIOVASCULAR events but do significantly lower BP in the hypertensive subgroup	[54–57, 60–64, 66, 69, 77, 78]

ARBs	Candesartan, valsartan, losartan, telmisartan	Nondialyzable	Effective in lowering BP and are recommended as first‐line agents for ESRD patients, particularly those with diabetes. ARBs offer a pharmacokinetic advantage over ACEIs because they are nondialyzable, maintaining more consistent drug levels during dialysis The HELD trial showed that losartan modestly but significantly lowered postdialysis systolic BP without increasing the risk of intradialytic hypotension or hyperkalemia Losartan improved postdialysis BP without increasing hypotension or hyperkalemia	Associated with significantly lower all‐cause mortality compared to non‐users Reduces heart failure events by 33% compared to placebo in dialysis patients A large cohort study showed ARB‐based therapy halved the risk of cardiovascular events compared to no ARB therapy in HD patients ARBs may offer a safer cardiovascular mortality profile compared to ACEIs when used in combination therapy settings	[53, 54, 56–58, 60–63, 65–69, 77, 80]

CCBs	Amlodipine, verapamil, cilnidipine	Nondialyzable	CCBs are the most commonly prescribed antihypertensives in HD patients and effectively reduce BP Both dihydropyridines (DHPs) and non‐dihydropyridines (non‐DHPs) are effective for BP control, but DHPs are preferred due to their favorable pharmacokinetics and nondialyzability CCBs, particularly DHPs, have a low incidence of serious side effects like bradyarrhythmias or intradialytic hypotension when appropriately dosed Newer agents like cilnidipine may improve ambulatory BP control and reduce BP variability in challenging dialysis cases	Amlodipine significantly reduces LVMI and improves endothelial function in hypertensive HD patients, suggesting added cardiovascular protective benefits Amlodipine reduced all‐cause mortality and cardiovascular events by 47% compared to placebo in hypertensive HD patients over 19 months A national U.S. cohort showed that CCB users had a 26% lower cardiovascular mortality and a 21% lower all‐cause mortality compared to non‐users	[40, 70–78]

Diuretics	Furosemide, hydrochlorothiazide, chlorthalidone	Nondialyzable	Diuretics are effective for BP control in HD patients, with studies showing significant reductions in systolic BP among users compared to non‐users Combination therapy using diuretics and other antihypertensives results in greater BP reduction than monotherapy in dialysis patients, suggesting synergistic benefits Continuation of loop diuretics in HD patients with residual renal function improves volume control, reduces interdialytic weight gain, and decreases the risk of intradialytic hypotension Thiazide diuretics remain effective in reducing BP even in patients with very low GFR (stage 3b–5 CKD)	The DOPPS study found that loop diuretic use was associated with a 14% lower risk of cardiac death and a nonsignificant 7% reduction in all‐cause mortality compared to non‐users No significant difference in 1‐year mortality between patients who continued vs. discontinued loop diuretics at dialysis initiation Continuation of diuretics was associated with lower rates of all‐cause hospitalization and reduced episodes of intradialytic hypotension, factors that could indirectly contribute to improved survival Evidence on mortality impact is inconsistent, and further high‐quality randomized controlled trials are needed to clarify the survival benefits of diuretics in the dialysis population	[79–90]

Direct vasodilators	Hydralazine, nitroprusside	Hydralazine is nondialyzable Nitroprusside is dialyzable	Hydralazine significantly reduces BP but requires careful monitoring for side effects like reflex tachycardia and fluid retention Isosorbide mononitrate alone reduced left ventricular hypertrophy and decreased acute heart failure events compared to placebo Combination hydralazine and nitrate therapy showed BP‐lowering benefits but had mixed results regarding cardiac remodeling Newer studies suggest that combination direct vasodilator therapy may benefit patients with refractory hypertension or left ventricular hypertrophy, although evidence remains limited	Hydralazine and isosorbide nitrate combination therapy did not significantly reduce mortality compared to placebo in dialysis patients Isosorbide mononitrate alone showed improved cardiovascular outcomes and lower acute heart failure events compared to placebo Some observational studies suggested potential cardiovascular benefits, but increased adverse events like symptomatic hypotension were noted Some studies suggest that hydralazine‐based therapy may help reduce left ventricular mass, a surrogate marker for better cardiovascular outcomes, but definitive mortality benefits have not been established	[92–95]

Alpha‐2‐agonist	Clonidine, Methyldopa, Guanfacine	Dialyzable Guanfacine is not dialyzable	Clonidine effectively lowers BP in dialysis patients, particularly for resistant hypertension Clonidine may help manage intradialytic hypertension when conventional therapies are insufficient Methyldopa is rarely used due to poor tolerability and is substantially removed by hemodialysis, with postdialysis recurrence of hypertension limiting sustained blood pressure control Guanfacine lowers blood pressure and has low dialysis clearance, but evidence in hemodialysis patients is limited, supporting use only as adjunctive therapy Side effects like dry mouth, bradycardia, and sedation can limit tolerability, potentially impacting adherence and outcomes	Observational studies suggest clonidine improves BP but have not shown consistent survival benefit No large randomized trials directly evaluating clonidine’s impact on mortality in dialysis patients are available Clonidine’s short‐term BP‐lowering effects have not been clearly linked to reductions in long‐term cardiovascular morbidity or mortality in dialysis patients Methyldopa has no evidence of cardiovascular or all‐cause mortality benefit in patients with ESRD on hemodialysis Guanfacine has not been shown to improve mortality or cardiovascular outcomes in CKD or hemodialysis populations, with available data limited to blood pressure effects	[92, 97–103].

MRAs	Spironolactone, finerenone, eplerenone	Nondialyzable	Spironolactone improves endothelial function and may reduce vascular stiffness in dialysis patients Low‐dose spironolactone has been shown to improve echocardiographic markers of cardiac structure without worsening hyperkalemia Eplerenone and finerenone (newer agents) are under investigation, but data in HD patients remain limited The risk of hyperkalemia remains a major concern and requires close monitoring during therapy but appears minimal in studies	Small trials show spironolactone reduces cardiovascular mortality in dialysis patients without significantly increasing hyperkalemia when carefully monitored Observational studies link aldosterone blockade with better survival rates in ESRD patients Use of mineralocorticoid receptor antagonists may provide additional cardiovascular protection beyond BP lowering	[104–113, 118]

Minoxidil		Dialyzable	Potent vasodilator that can significantly reduce BP in resistant hypertension cases among HD patients, although careful monitoring for fluid retention and tachycardia is required Minoxidil’s use requires combination with diuretics and beta‐blockers to manage side effects like fluid retention and reflex tachycardia It can lead to significant reductions in left ventricular hypertrophy due to afterload reduction	No large RCTs have assessed the direct impact of minoxidil on mortality in dialysis patients Observational data suggest that while minoxidil controls BP effectively, it may be associated with higher hospitalization rates due to fluid overload if not used with diuretics Minoxidil is primarily used for BP control rather than mortality benefit, and it should be reserved for patients with severe resistant hypertension	[114–117]

Further research through large‐patient RCTs is imperative to develop definitive guidelines for selecting, dosing, timing, and combining antihypertensive therapies in dialysis patients. Individualizing treatment based on patient comorbidities, residual renal function, volume status, and hemodynamic stability is critical to balancing efficacy and safety. Addressing the unresolved questions surrounding antihypertensive use in this population should remain a top research priority, given the dismal cardiovascular prognosis of dialysis patients.

## Funding

We declare this research received no funding.

## Disclosure

No financial, personal, or other relationships influenced this study’s design, conduct, interpretation, or publication.

## Conflicts of Interest

The authors declare no conflicts of interest.

## Data Availability

Data sharing is not applicable to this article as no datasets were generated or analyzed during the current study.
